# Fault Diagnosis of Induction Machines in a Transient Regime Using Current Sensors with an Optimized Slepian Window

**DOI:** 10.3390/s18010146

**Published:** 2018-01-06

**Authors:** Jordi Burriel-Valencia, Ruben Puche-Panadero, Javier Martinez-Roman, Angel Sapena-Bano, Manuel Pineda-Sanchez

**Affiliations:** Institute for Energy Engineering, Universitat Politècnica de València, Cmno. de Vera s/n, 46022 Valencia, Spain; jorburva@die.upv.es (J.B.-V.); jmroman@die.upv.es (J.M.-R.); asapena@die.upv.es (A.S.-B.); mpineda@die.upv.es (M.P.-S.)

**Keywords:** fault diagnosis, condition monitoring, short time Fourier transform, Slepian window, prolate spheroidal wave functions, discrete prolate spheroidal sequences, time-frequency distributions

## Abstract

The aim of this paper is to introduce a new methodology for the fault diagnosis of induction machines working in the transient regime, when time-frequency analysis tools are used. The proposed method relies on the use of the optimized Slepian window for performing the short time Fourier transform (STFT) of the stator current signal. It is shown that for a given sequence length of finite duration, the Slepian window has the maximum concentration of energy, greater than can be reached with a gated Gaussian window, which is usually used as the analysis window. In this paper, the use and optimization of the Slepian window for fault diagnosis of induction machines is theoretically introduced and experimentally validated through the test of a 3.15-MW induction motor with broken bars during the start-up transient. The theoretical analysis and the experimental results show that the use of the Slepian window can highlight the fault components in the current’s spectrogram with a significant reduction of the required computational resources.

## 1. Introduction

Rotating electrical machines cover a broad range of applications in modern industrial installations. Particularly, cage induction machines are the most widely used due to their robustness and low maintenance requirements. Ensuring their proper functioning is essential to keep the production processes running [1]. Thus, the early detection of induction machine (IM) faults and the machine condition prognosis are crucial to reduce maintenance costs [2] and to avoid costly, unexpected shut-downs [3]. Fault diagnosis via the current analysis in the frequency domain has become a common method for machine condition evaluation because it is non-invasive, it requires a single current sensor, either a current transformer, a Hall sensor or a magnetoelectric current sensor [4], and it can identify a wide variety of machine faults [5,6]. Traditionally, these techniques, known as motor current signature analysis (MCSA), have focused on the detection of faults during the steady state functioning of the machine through the current spectrum, which can be computed using the fast Fourier transform (FFT) [7,8,9,10]. For example, bar breakages in the rotor cage produce components of frequencies $f_{bb}$ [9,11,12,13,14,15,16];

(1) $$f_{bb}=\left|\left(1\pm 2ks\right)\right|f_{supply}\;k=1,2,3\ldots ,$$

a mixed eccentricity fault generates components of frequencies $f_{ecc}$ [17,18,19];

(2) $$f_{ecc}=\left|\left(1\pm k\frac{1-s}{p}\right)\right|f_{supply}\;k=1,2,3\ldots ,$$

and bearing faults generate components of frequencies $f_{bear}$ [20,21,22];

(3) $$f_{bear}=\left|\left(1\pm kf_{0}\right)\right|\;k=1,2,3\ldots ,$$

where *s* is the slip, $f_{supply}$ is the frequency of the power supply, *p* is the number of pole pairs and $f_{o}$ corresponds to one of the characteristic vibration frequencies generated by the bearing fault, which depends on the bearing dimensions and on the mechanical rotor frequency [8]. However, in many applications, the slip, the supply frequency and the mechanical rotor frequency can be variable, which render traditional MCSA techniques inadequate for fault diagnosis of electrical machines working in non-stationary conditions. Examples of such transient conditions are the start-up transients, continuous changes in load or speed [23] or the use of a variable frequency supply, especially in machines fed through variable speed drives (VSD). This inadequacy resides in the FFT being unsuitable to identify fault frequencies that are no longer constant.

To extend MCSA to such working conditions, recently, transient MCSA (TMCSA) techniques have been developed using different approaches. One approach relies on using only time-domain features to isolate and to detect the fault: first, the fault components of the current are extracted, using a band-pass filter tailored to the frequency band spanned by the fault harmonics during the transient conditions of the machine; and second, the RMS value of these components is used to detect the fault. In [24,25], empirical mode decomposition (EMD) is used to extract the fault components. In [26], the recursive undecimated wavelet packet transform (RUWPT) is used to isolate and to compute the RMS value of the components produced by a broken bar fault, using an extremely low sampling frequency (224 Hz) and a small number of current samples (1024 samples). Other approaches rely on tracking the evolution of the fault harmonics in the time-frequency domain, looking for characteristic patterns of each type of fault, as indicated by Equations (1)–(3); this technique allows the detection of different types of faults, even in the case of mixed faults, with the instantaneous presence of two faults, such as broken rotor bars in the presence of the intrinsic static eccentricity; as [27] states, rotor bars’ breakage causes static eccentricity, and it is possible that two faults occur simultaneously. TMCSA techniques have been developed in the technical literature using different time-frequency (TF) signal analysis tools [9,28], such as the discrete wavelet transform (DWT) [12,15,19,29,30,31,32,33], the discrete wavelet packet transform (DWPT) [34], the discrete harmonic wavelet transform (DHWT) [35], the continuous wavelet transform (CWT) [36,37], the complex CWT [38,39] and the Wigner-Ville distribution (WVD) [40,41], among others. Wavelet-based transforms require a proper choice of the mother wavelet and a precise adjustment of the sampling frequency and the number of bands of the decomposition to perform fault diagnosis. Quadratic-based transforms, such as the WVD, have, as their main drawback, the appearance of the cross-term effects that can smear the spectrogram of the current signal. The minimization of cross-term effects has been widely discussed in the technical literature [40,41,42,43,44]. However, in the case of the STFT [41,45], which can be considered the natural extension of FFT-based MCSA techniques, the cross-term effects do not appear, as the STFT is a linear transform. The STFT, as the WVD, can obtain a TF distribution with enough resolution to discriminate the different harmonic components of the signal, but without cross-term effects [3]. Thus, a STFT-based approach is proposed in this paper.

The STFT is defined as [46]

(4) $$S_{f}\left(t,\omega \right)=\int i\left(\tau \right)g\left(t-\tau \right)e^{-j\omega \tau }d\tau ,$$

where i(t) is the stator current and g(t) is the analysis window. The spectrogram $P_{SP}\left(t,\omega \right)$ is given by

(5) $$P_{SP}\left(t,\omega \right)={\left|S_{f}\left(t,\omega \right)\right|}^{2},$$

which can be re-written as [47]

(6) $$P_{SP}\left(t,\omega \right)=\frac{1}{2\pi }\int \int W_{i}\left(\tau ,\nu \right)W_{g}\left(\tau -t,\nu -\omega \right)d\tau d\nu ,$$

where $W_{i}\left(t,\omega \right)$ and $W_{g}\left(t,\omega \right)$ are the WVD of the stator current and the analysis window, respectively. Thus, the spectrogram can be considered as the 2D smoothing of the WVD of the current signal by the WVD of the analysis window [48]. In other words, the window involves smoothing the oscillatory interference between individual components, which appears due to the quadratic nature of the WVD. Hence, the window must be selected with the aim of highlighting the TF information of the analyzed signal and, at the same time, with the goal of reducing to a minimum the smearing of the spectrogram [49]. In fact, the optimal window is the one that, for a given total duration, maximizes the amount of the total energy in a given bandwidth. However, as the uncertainty principle states, one cannot construct any signal for which both the standard deviation in time, $\sigma_{t}$, and the standard deviation in frequency, $\sigma_{\omega }$ (i.e., the duration and the bandwidth), are arbitrarily small [50]. In fact, the minimum achievable values of $\sigma_{t}$ and $\sigma_{\omega }$ must satisfy Heisenberg’s inequality [50]:

(7) $$\sigma_{t}\cdot \sigma_{\omega }\geq 0.5.$$

The equality in Equation (7) is only achieved by the Gaussian pulse of infinite length [51]. However, real-world signals have a finite duration, and a gated Gaussian window is often not a good choice, as stated in [52]. In fact, in fault diagnosis methods for IMs, the current is sampled during a limited time, so it is a time-limited signal. However, besides, due to the limited bandwidth of the measurement channels, the current signal is also a band-limited signal. Unfortunately, the uncertainty principle tells us that a signal cannot be simultaneously time- and band-limited. A natural assumption is thus to consider mathematically the current signal as an almost time- and almost band-limited signal, in the way proposed in [53,54]; that is, using the model [55] of band-limited, or almost band-limited, functions that are sufficiently concentrated in time to represent both the current signal and the window used to analyze it.

Therefore, under this model: Which is the optimal window? Thanks to the work presented in [56,57,58], the optimal orthogonal system for representing almost time- and almost band-limited functions is known. This system consists of the so-called Slepian functions, also known as prolate spheroidal wave functions (PSWFs), which have two remarkable properties that make them optimal for being used as STFT windows:

- The Slepians are the band-limited functions that are the most concentrated in a fixed time interval in the $L^{2}$-norm [59]. Therefore, they can be considered as the optimal window for TF analysis of non-stationary currents [60], because they can highlight the energy content of the current signal in the joint time-frequency domain with the highest possible resolution among all the almost time- and band-limited windows, including the truncated Gaussian window.
- Alternatively, the Slepians can be considered as the time-limited functions that are the most concentrated in a fixed frequency interval in the $L^{2}$-norm. That is, for a given bandwidth, they are the shortest possible windows that can be used for generating the current spectrograms, which allows the reduction of the time needed to build such spectrograms.

Both properties, the increase of the resolution of the current spectrogram and the reduction of the computing time needed to obtain it, will be assessed in the Experimental Section of this paper. The Slepian windows have been used in other fields such as medical image diagnostics [61], wireless transmission [62], acoustics [63], signal processing [64], etc. However, in spite of their benefits, to the best knowledge of the authors, they have never been used before for the fault diagnosis of IMs through the analysis of the stator current.

Therefore, the main goals of this work are, first, to introduce theoretically the Slepian window; second, to demonstrate its suitability for the fault diagnosis of electrical machines; and finally, to provide criteria for optimizing the parameters of the Slepian window depending on the type of the diagnosed fault. The broken bar fault is used in this paper to present the application of the Slepian window for the fault diagnosis of IMs, without any loss of generality, because the proposed method is valid for the diagnosis of any IM fault that generates a characteristic series of harmonics in the stator current, such as Equations (1)–(3).

This paper is structured as follows: in Section 2, the Slepian window is theoretically introduced and compared with the Gaussian window in terms of energy concentration. Section 3 presents the proposed procedure for using the Slepian window for fault diagnosis; to illustrate this method, it is applied to a synthetic signal simulating the evolution of the left sideband harmonics (LSH) produced by a broken bar during the start-up transient of an IM. In Section 4, the proposed approach is validated using a high-power, high-voltage IM with a broken rotor bar fault. In Section 5, the practical advantages of the proposed method are highlighted. In this section is proposed the use of a truncated Slepian window, which is able to display the evolution of the fault harmonics in the TF domain correctly with a huge reduction of the computational resources needed to obtain the spectrogram. In Section 6, the main conclusions of this work are presented.

## 2. The Slepian Functions for Fault Diagnosis of Rotating Electrical Machines in the Transient Regime

From Equation (6), it can be seen that the analysis window has a major effect on the spectrogram of the current. It highlights the harmonic components of the current, but at the same time, it smears the spectrogram (6), so it has a major impact on the reliability of the fault diagnostic procedure. The election of a window maximally confined to a region of the TF plane with a limited duration and bandwidth is crucial to obtain a high resolution spectrogram, which accurately reflects the fault components of the current in the TF plane, with a minimum of smearing due to the use of the window. Therefore, the spectrogram obtained with this optimal window can improve the diagnostic decision process, compared with the use of non-optimal windows. The type of windows that are optimally and maximally concentrated, for a finite duration and bandwidth, are the Slepians [58,65]. Accordingly, in this paper, the Slepian window is proposed for the fault diagnosis of IMs. In the following subsections, its characteristics and the procedure to adjust its parameters are presented.

### 2.1. Theoretical Introduction to the Slepian Functions

The Slepians functions are defined [52,66,67] as the solutions of the integral Equation

(8) $$\int_{-T}^{T}\varphi \left(x\right)\frac{\sin B(t-x)}{\pi (t-x)}dx=\lambda \varphi \left(t\right)$$

for eigenvalues $\lambda =\lambda_{n}$. There are infinite eigenvalues, all of them real numbers, positive and smaller than 1,

(9) $$1>\lambda_{0}>\lambda_{1}>\cdots >\lambda_{n}>\cdots >0.$$

The integral Equation (8) states that trimming the Slepian function of order *n*, $\varphi_{n}\left(t\right)$, with a rectangular window in the [−T,T] interval, will reproduce $\varphi_{n}\left(t\right)$, except for a factor $\lambda_{n}$. Besides, the convolution kernel sin(Bt)/πt in Equation (8) represents a sharp low-pass filtering process in the frequency domain. Hence, $\varphi_{n}\left(t\right)$ is a low-pass function with almost no energy at angular frequencies outside the interval [−B,B].

The Slepians have the remarkable property of orthogonality, both over an infinite and a finite range of the independent variable [65]. Due to the fact that the functions $\varphi_{n}\left(t\right)$ form a complete set of orthonormal functions, band-limited functions y(t) can be expanded in terms of the Slepians with the same bandwidth as

(10) $$y\left(t\right)=\sum_{k=0}^{\infty }a_{k}\varphi_{k}\left(t\right),$$

where

(11) $$a_{k}=\int_{-\infty }^{\infty }y\left(t\right)\varphi_{k}\left(t\right)dt.$$

Another remarkable property of the Slepians is that, as the Gaussian functions, each Slepian function, $\varphi_{n}\left(t\right)$, is proportional to its Fourier transform (FT), ${\hat{\varphi }}_{n}\left(\omega \right)$, in a finite interval

(12) $${\hat{\varphi }}_{n}\left(\omega \right)\approx \varphi_{n}\left(t=\frac{T}{B}\omega \right)\;\mathrm{for}\;\left|\omega \right|<B,$$

where *T* is half of the total duration and *B* is the positive bandwidth (in rad/s), equal to half of the total bandwidth. Using Equations (10) and (12), a time-limited signal y(t) can be expanded in terms of the FT of the functions $\varphi_{k}\left(t\right)$, ${\hat{\varphi }}_{k}\left(\omega \right)$, which vanish for −T<t<T

(13) $$y\left(t\right)=\sum_{k=0}^{\infty }b_{k}{\hat{\varphi }}_{k}\left(\frac{B}{T}t\right).$$

where

(14) $$b_{k}=\int_{-\infty }^{\infty }y\left(t\right)\varphi_{k}\left(t=\frac{T}{B}\;\omega \right)dt.$$

The main application of the Slepian functions is the design of band-limited signals with a maximum energy concentration in a given time and frequency interval. In the next subsections, the energy concentration of a Slepian window for a given duration and bandwidth is obtained, first separately in each domain and, afterwards, in the joint TF domain.

### 2.2. Energy of the Slepian Windows in a Time Interval

Given a band-limited signal, y(t), it can be expanded into the properly scaled functions $\varphi_{k}\left(t\right)$ in Equation (10). Taking into account the orthonormality of the Slepian functions [52]

(15) $$\int_{-\infty }^{\infty }\varphi_{k}\left(t\right)\varphi_{j}\left(t\right)dt=\left\{\begin{array}{ccc} 1 & \mathrm{if} & k=j\\ 0 & \mathrm{if} & k\neq j \end{array}\right.$$

the total energy *E* of the signal can be computed as

(16) $$E=\int_{-\infty }^{\infty }{\left|y\left(t\right)\right|}^{2}dt=\sum_{k=0}^{\infty }a_{k}^{2}.$$

The energy of the signal y(t) contained in the time interval of duration (−T,T), $E_{T}$, is given by

(17) $$E_{T}=\int_{-T}^{T}{\left|y\left(t\right)\right|}^{2}dt=\sum_{k=0}^{\infty }\lambda_{k}a_{k}^{2}.$$

From (16) and (17), the energy fraction $\alpha =E_{T}/E$ is

(18) $$\alpha =\frac{\sum_{k=0}^{\infty }\lambda_{k}a_{k}^{2}}{\sum_{k=0}^{\infty }a_{k}^{2}}.$$

Therefore, the band-limited window, which is maximally concentrated to a time interval (−T,T) is given by the maximum value of the ratio in Equation (18). Since $\lambda_{0}$ is greater than any other $\lambda_{k}$, this is achieved by setting all $a_{k}$ except $a_{0}$ equal to zero [52]. Hence, $\alpha_{max}=\lambda_{0}$, where $\lambda_{0}$ depends on the time-bandwidth product (B·T). For example, if B·T=1, then α≈0.6. On the contrary, if α is required to be as high as 0.95, then B·T≈3 [57,58]. Therefore, among all the band-limited functions with the same bandwidth, the zero order Slepian function, $\varphi_{0}\left(t\right)$, is the maximally concentrated one for a given duration.

### 2.3. Energy of the Slepian Windows in a Frequency Interval

The energy of the signal y(t) contained in the frequency interval of bandwidth (−B,B), $E_{B}$, is given by

(19) $$E_{B}=\int_{-B}^{B}{\left|\hat{y}\left(\omega \right)\right|}^{2}d\omega ,$$

and, applying Equations (13) and (14), the energy fraction $\beta =E_{B}/E$ is equal to

(20) $$\beta =\frac{\sum_{k=0}^{\infty }\lambda_{k}b_{k}^{2}}{\sum_{k=0}^{\infty }b_{k}^{2}}.$$

As done in the previous subsection, since $\lambda_{0}$ is greater than any other $\lambda_{k}$, the maximum ratio in Equation (20) is achieved by setting all $b_{k}$ except $b_{0}$ equal to zero [52]. Therefore, among all the time-limited functions with the same duration, the zero order Slepian function, $\varphi_{0}\left(t\right)$, is the maximally concentrated one for a given bandwidth.

### 2.4. Energy of the Slepian Windows in the Joint TF Domain

As can be deduced from Equations (18) and (20), the largest energy concentration both in the time and in the frequency domains, considered independently, is achieved by the zero order Slepian function, $\varphi_{0}\left(t\right)$. Similarly, in the joint TF domain, the zero order Slepian function is also the function with the largest possible product of energy fractions, α·β, which is obtained for α=β, as in [52]

(21) $${\left(\alpha \cdot \beta \right)}_{max}={\left(\frac{1+\sqrt{\lambda_{0}}}{2}\right)}^{2}.$$

### 2.5. Comparison between the Slepian Window and the Gaussian Window

The Gaussian window g(t) is defined as [46]

(22) $$g\left(t\right)={\left(\frac{\gamma }{\pi }\right)}^{1/4}e^{-\frac{\gamma t^{2}}{2}},$$

being

(23) $$\gamma =\frac{1}{2\sigma_{t}^{2}}\;.$$

As in the case of the Slepian window, the FT of the Gaussian window, $\hat{g}\left(\omega \right)$, is a scaled version of itself [46]

(24) $$\hat{g}\left(\omega \right)={\left(\frac{1}{\gamma \pi }\right)}^{1/4}e^{-\frac{\omega^{2}}{2\gamma }}\;,$$

where

(25) $$\gamma =2\sigma_{\omega }^{2}\;.$$

The Gaussian window of infinite length is optimal in terms of minimization of Equation (7), but for a finite duration and for a given bandwidth, the zero order Slepian function achieves the maximum concentration of energy in the joint TF domain. For example, for $\lambda_{0}=0.6$, (B·T≈1), the product of energy fractions (21) is ${\left(\alpha \cdot \beta \right)}_{max}=0.787$ in the case of the Slepian window. The Gaussian window has infinite length and infinite bandwidth, so for computing the energy fractions α and β, the values of half of the total duration *T* and half of the total bandwidth *B* have been chosen as the values of the respective standard deviations, as in [52]. That is, $T=\sigma_{t}$ and $B=\sigma_{\omega }$. With these settings, the product (α·β) for the Gaussian window is only about 0.466 [52].

**Figure 1:**
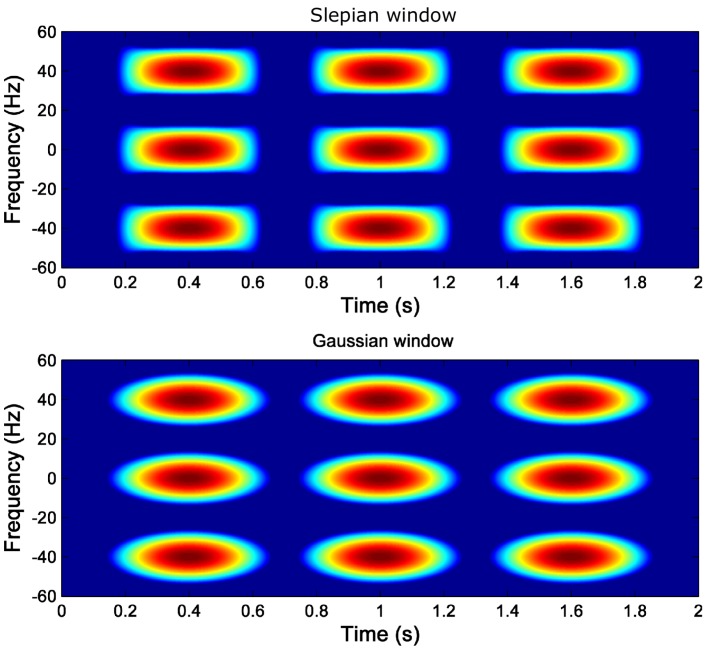
Time-frequency atoms of a Slepian window (**top**) and of a Gaussian window (**bottom**).

Figure 1 shows the Heisenberg boxes of the Slepian and of the Gaussian atoms in the TF plane. The Slepian atom has a rectangular shape, while the Gaussian atom extends radially from its center. Besides, the rectangular shape of the Slepian atom allows an efficient tiling of the TF domain and is specially well suited for the proposed diagnostic approach, just by choosing the diagonal of the Slepian window to be parallel to the fault component trajectory in the TF plane [51], as will be developed in the next subsection.

### 2.6. Proposed Method for the Choice of the Parameters of the Slepian Window

**Figure 2:**
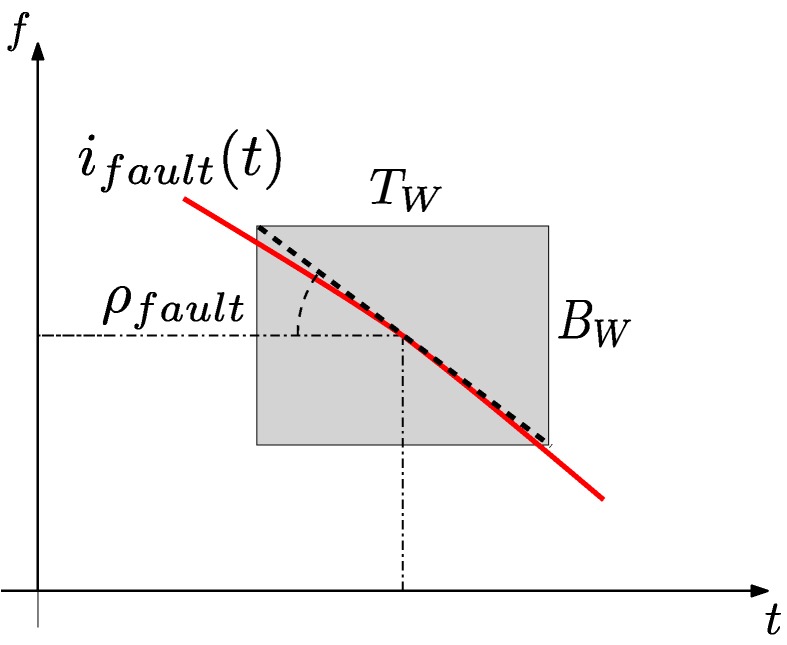
Choice of the parameters of the Slepian window so that the aspect ratio of its Heisenberg box coincides with the slope of the trajectory of the related fault component in the time-frequency (TF) plane.

In this subsection, the method for selecting the parameters that optimize the Slepian window for detecting a given fault is presented. As the frequencies of the different faults in Equations (1)–(3) are given in Hz, it is advisable to define this optimal window using its total bandwidth expressed in Hz, that is $B_{W}=\frac{2B}{2\pi }=\frac{B}{\pi }$. Besides, the implementation of the STFT algorithms relies on the length of the window, so it is advisable also to characterize the Slepian window using its total duration in seconds, $T_{W}=2T$, as depicted in Figure 2.

Based on the characteristics of the Slepian window in terms of energy concentration in a limited time-frequency region, the first criterion to determine the window parameters is to establish the maximum energy concentration desired for the window, ${\left(\alpha \cdot \beta \right)}_{max}$, which imposes the time-bandwidth product, $B_{W}\cdot T_{W}$. In this paper, an energy concentration as high as possible is proposed, i.e., (α·β)≈1, which, from Equation (21), gives $\lambda_{0}\approx 1$. According to [57], this can be obtained with a time-bandwidth product $B_{W}\cdot T_{W}=8$

(26) $${\left(\alpha \cdot \beta \right)}_{max}\approx 1\to \lambda_{0}\approx 1\to B_{W}\cdot T_{W}=8$$

However, there are infinite combinations of $B_{W}$ and $T_{W}$ that meet condition Equation (26), so an additional criterion is needed to establish both $B_{W}$ and $T_{W}$. These two parameters can be selected according to different criteria. In [68], the optimal bandwidth of the window for signals with time-varying frequency is found to be equal to the square root of the derivative of the instantaneous frequency (IF) of the signal. In [51,69], the optimal parameters of the Gaussian window are those that minimize the TF area occupied by a target component. To achieve this optimization, in this work, the Slepian window is selected to have the maximum overlap with the trajectory of the fault harmonic signal in the TF plane, as in [51,70]. This condition is met when the magnitude of the slope of this trajectory, $\rho_{fault}$, and the aspect ratio $B_{W}/T_{W}$ of Heisenberg’s box of the Slepian window coincide (Figure 2), so that

(27) $$\frac{B_{W}}{T_{W}}=\rho_{fault}=|\frac{d(f_{fault}\left(t\right))}{dt}|.$$

Hence, combining Equations (26) and (27), the two conditions proposed for selecting the optimal parameters of the Slepian window are

(28) $$\left.\begin{array}{ccc} B_{W}\cdot T_{W} & = & 8\\ \frac{B_{W}}{T_{W}} & = & \rho_{fault} \end{array}\right\}$$

From Equation (28), the optimal length of the Slepians window is given by

(29) $$T_{W}=\sqrt{\frac{8}{\rho_{fault}}}$$

which is valid for any type of fault. For example, $\rho_{fault}$ can be computed from Equation (1)–(3) for the detection of a broken rotor bar, mixed eccentricity and bearing faults, respectively. In the following sections, the proposed approach has been applied to the diagnosis of broken rotor bars, as in [9,11,12,13,15,71], without any loss of generality. In this case, $\rho_{fault}$ is calculated as the derivative of Equation (1) with respect to time. Taking into account that $s=\frac{n_{s}-n}{n_{s}}$, where *n* is the mechanical speed of the rotor (rpm) and $n_{s}=60f_{supply}/p$ is the synchronous speed of the machine, this derivative gives, in the case of constant $f_{supply}$,

(30) $$\rho_{fault}=|\frac{d(\left(1\pm 2ks\right)f_{supply})}{dt}|=2kf_{supply}|\frac{ds}{dt}|=\frac{2kf_{supply}}{n_{s}}|\frac{dn}{dt}|=\frac{kp}{30}|\frac{dn}{dt}|\;k=1,2,3\ldots$$

That is, the slope of the broken bar fault harmonic at every time instant is simply the acceleration of the machine at that instant, up to a constant scale factor.

The slope of the trajectory of the fault harmonic in the TF plane is computed at the center of the Slepian window, shown in Figure 2, as in [72]. Assuming a low variation of the IF of the fault harmonic during the short duration of the window, a first order, linear approximation of this trajectory can be used, as in [73]. In case of long-term variations of the IF, the original current signal can be divided into a number of time segments where this approximation can be applied, as suggested in [72,74].

The practical implementation of the proposed method is very simple with modern computing software. Effective algorithms for computing the Slepian window can be found in [75]. In MATLAB, there is a function that returns a Slepian sequence named dpss (discrete prolate spheroidal sequences), which can be called

(31) dps_seq=dpss(seq_length, time_halfbandwidth, 1,

where seq_length is the length of the Slepian window in samples and time_halfbandwidth is equal to $B_{W}\cdot T_{W}/2$. Applying Equations (28) and (29) to Equation (31), the optimum Slepian window for detecting a given fault is obtained easily as

(32) $$\mathrm{dps}\_\mathrm{seq}=\mathrm{dpss}(\mathrm{round}\left(f_{sampling}\times \sqrt{\frac{8}{\rho_{fault}}}\right),\;4,\;1),$$

when using a sampling frequency $f_{sampling}$.

## 3. STFT of the Start-Up Current of a Simulated IM Using the Slepian Window

In this section, the use of a Slepian window for the analysis of the current through the STFT is presented, and it is illustrated using the LSH generated during the start-up of a simulated machine with a broken rotor bar, whose main characteristics are given in Appendix A. The simulation has been performed during 2 s using a sampling frequency of 5 kHz, giving a total of 10,000 samples.

### 3.1. Evolution of the LSH during the Start-Up Transient of an IM

The evolution of the LSH of an IM with a broken bar during the start-up transient has been analyzed in [9,15,76,77]. In this work, the LSH evolution is extracted from the current signal of a simulated machine. Basically, the LSH fault component is a sinusoidal signal whose amplitude and frequency vary continuously depending on the slip *s*.

**Figure 3:**
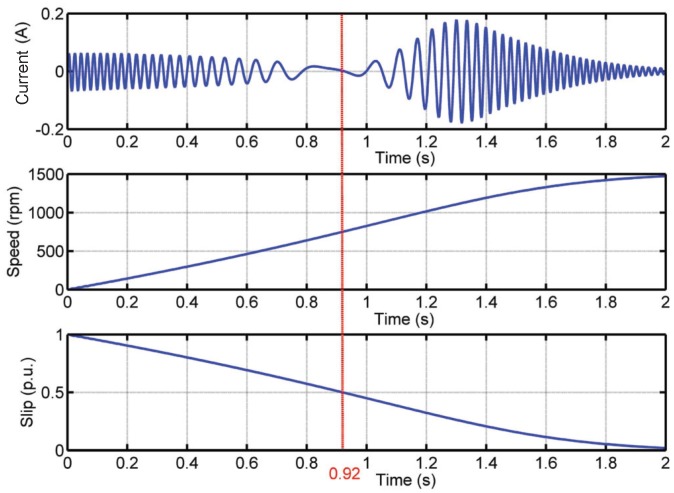
Time evolution of the left sideband harmonic (LSH) (**top**), of the motor speed (**middle**) and of the motor slip (**bottom**) during the start-up transient of the simulated induction machine (IM) given in Appendix A. The vertical line corresponds to the time when the slip s=0.5 is reached.

The LSH amplitude (Figure 3) follows a characteristic evolution. First, the amplitude decreases until it disappears (slip s=0.5, time t=0.92s). During the second half of the start-up transient (t>0.92s), the amplitude increases up to a maximum value and, after, decreases towards its steady-state value.

**Figure 4:**
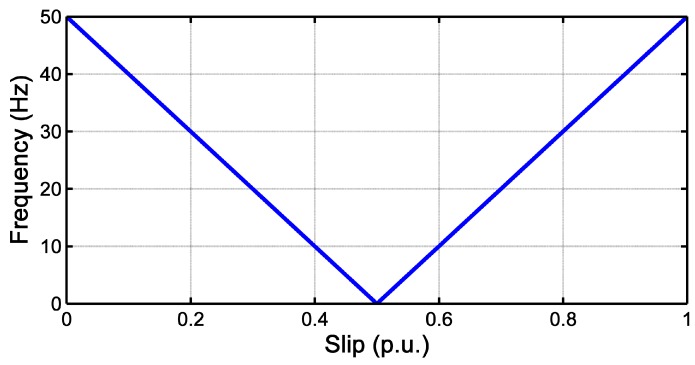
Evolution of the frequency of the LSH as a function of the rotor slip.

The frequency of the LSH varies as shown in Figure 4. The initial frequency of the LSH, at s=1, is the same as the supply frequency ($f_{supply}=50$ Hz), and after, it decreases, becoming null when the rotor slip is equal to 0.5. From this point, the frequency of the LSH increases again, keeping a constant value (slightly below the supply frequency) when the steady-state regime is reached.

**Figure 5:**
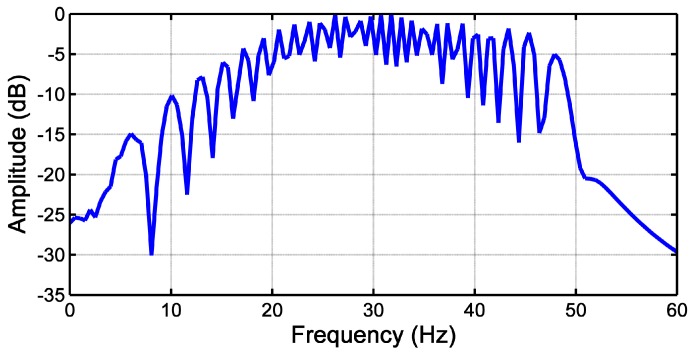
Spectrum of the LSH.

Traditional MCSA methods cannot be used for the diagnosis of this fault in the transient regime. In the spectrum of the LSH shown in Figure 5, there is no peak signaling the presence of LSH, because its frequency is not constant. Hence, the FFT cannot properly highlight the TF evolution of the fault harmonic component generated in the stator current by the fault.

### 3.2. Choice of the Parameters of the Slepian Window

The aim of this section is to build a Slepian window suitable for identifying the LSH during the start-up transient of the IM. As deduced in Section 2.6, this implies calculating the parameters $B_{W},T_{W}$ from Equation (28), and consequently, a value of $\rho_{fault}$ has to be adopted. In this work, the value of $\rho_{fault}$ in Equation (28) will be taken as its average value during the start-up transient. This is a reasonable assumption whenever the acceleration of the machine during the start-up is quite regular, as happens if the inertia factor is not very low (see Figure 3). An approximated value of the averaged value of $\rho_{fault}$ for the LSH is obtained fromEquation (30), taking k=1,

(33) $$\rho_{fault}\approx 2f_{supply}{|\;\frac{\Delta s}{\Delta t}|\;}_{s=1}^{s=0.5}=2f_{supply}|\frac{0.5-1}{t_{s=0.5}-0}|=\frac{f_{supply}}{t_{s=0.5}}\;,$$

or, also,

(34) $$\rho_{fault}\approx 2\frac{f_{supply}}{n_{s}}{|\;\frac{\Delta n}{\Delta t}|\;}_{n=0}^{n=n_{s}}\approx \frac{f_{supply}}{t_{startup}/2}\;,$$

where $t_{s=0.5}$ is the time that the rotor takes to reach half of the synchronous speed, and $t_{startup}$ is the start-up time. Therefore, the maximum overlapping conditions Equations (28) and (33) are combined with the level of maximum energy concentration Equation (26), giving

(35) $$\left.\begin{array}{ccc} B_{W}\cdot T_{W} & = & 8\\ \frac{B_{W}}{T_{W}} & = & \rho_{fault}=\frac{f_{supply}}{t_{s=0.5}} \end{array}\right\}$$

**Figure 6:**
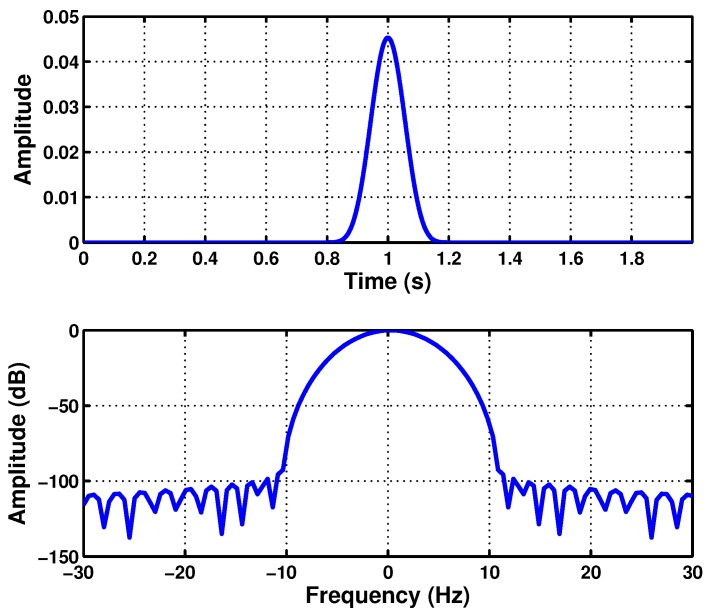
Slepian window ($B_{W}=20.85$ Hz, $T_{W}=383.7$ ms) optimized for the maximum overlap with the LSH trajectory in the time domain (**top**) and in the frequency domain (**bottom**).

**Figure 7:**
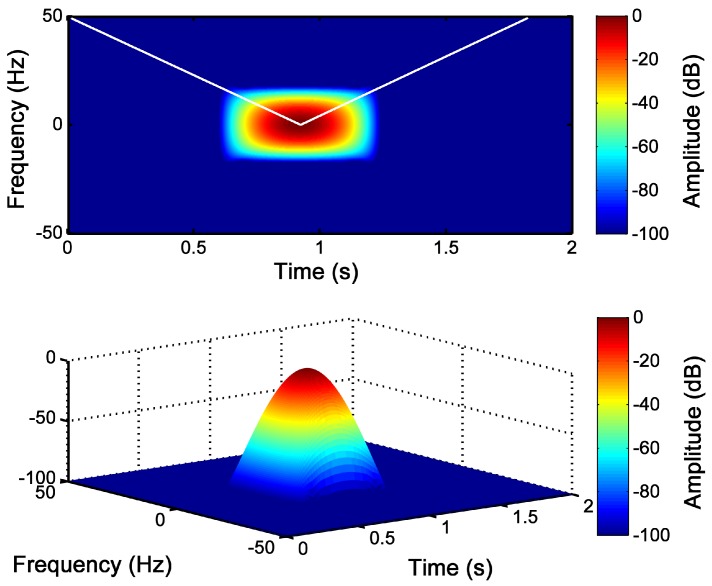
Slepian window ($B_{W}=20.85$ Hz, $T_{W}=383.7$ ms) optimized for representing the LSH, as a 2D view (**top**) and as a 3D view (**bottom**) in the time-frequency plane. The white line marks the trajectory of the LSH in this plane.

In this case, for the simulated machine, from Figure 3, $t_{s=0.5}=0.92$ s, and thus, $B_{W}/T_{W}=50/0.92=54.35$ Hz/s. Therefore, the parameters of the optimal Slepian window are $B_{W}=20.85$ Hz and $T_{W}=383.7$ ms. This window is represented in separated into the time and frequency planes in Figure 6, located at the center of the respective domains. Almost all the energy of the window is concentrated under the main lobe of the window in the frequency domain. On the other hand, in Figure 7, the designed Slepian window has been represented in the TF plane, in two and three dimensions. Moreover, the slope of the LSH has been superimposed (white line) in Figure 7, showing that the designed window is optimal for this signal, because it achieves the maximum overlapping with the fault component trajectory in the TF plane.

The assumption of linear instantaneous frequency during the start-up transient is quite accurate in the case of large IMs (for which the condition monitoring is especially interesting) or IMs driving constant loads. In case of non-linear instantaneous frequency (IF) during the start-up, the total starting time can be sliced into time intervals with nearly constant IF slope (a first order approximation), as done in [78]. During each one of these time intervals, the procedure for selecting the parameters of the Slepian windows presented in this section can be applied, taking the value of $\rho_{fault}$ in Equation (28) as its average value in the interval.

### 3.3. Detection of the LSH Fault Component with the Slepian Window

**Figure 8:**
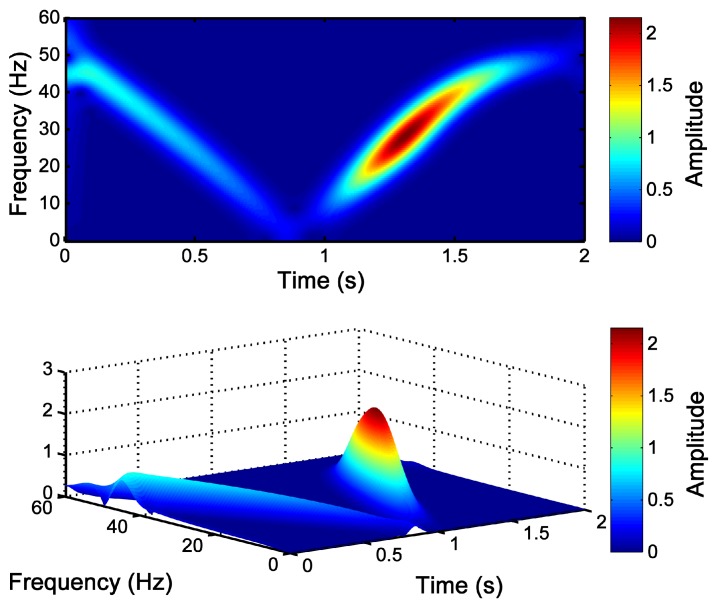
Time-frequency-amplitude pattern generated by the LSH obtained with the optimized Slepian window ($B_{W}=20.85$ Hz, $T_{W}=383.7$ ms), as a 2D view (**top**) and as a 3D view (**bottom**).

Once the window parameters have been selected using Equation (35), the Slepian window has been applied to obtain the STFT of the LSH fault component shown in Figure 3. As is shown in Figure 8, a high resolution image of the TF pattern of the LSH (Figure 4) has been obtained with this window. Besides, a linear scale has been used to represent the LSH spectrogram, so that the amplitude evolution of the LSH is visible. Initially, its amplitude decreases until it becomes null (s=0.5, $t_{s=0.5}=0.92$ s). During the second half of the start-up, the amplitude increases reaching a maximum, and finally, it decreases again towards the steady-state value. Therefore, the generated pattern can identify not only the instantaneous frequency of the LSH, but also its instantaneous amplitude, improving the reliability of the fault diagnosis process.

**Figure 9:**
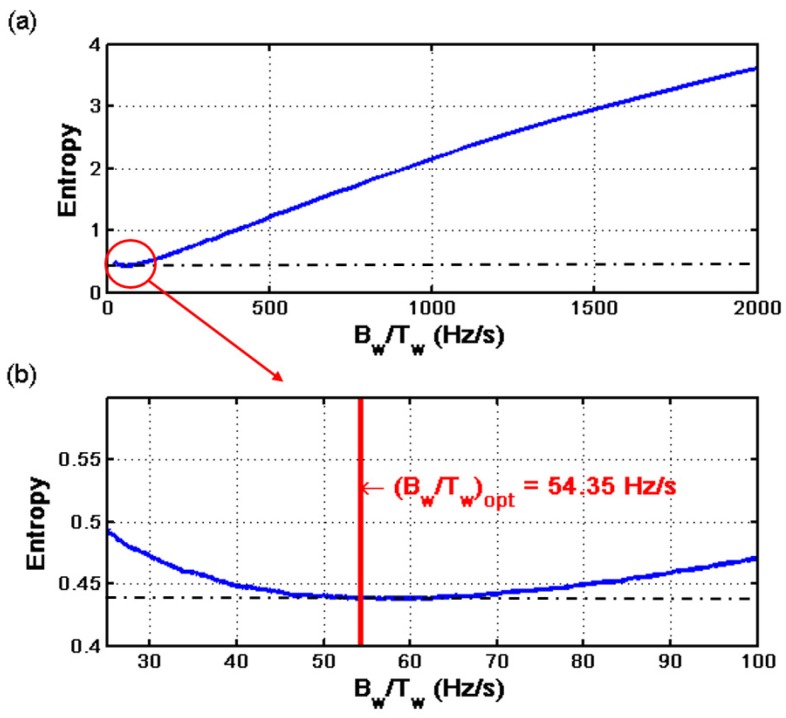
(**a**) Entropy of the time-frequency analysis of the LSH using the Slepian window, as a function of the parameter $B_{W}/T_{W}$; (**b**) zoomed area of the entropy in the interval close to the optimum value of $B_{W}/T_{W}$. The vertical line corresponds to the minimum entropy value, which coincides with the criteria of maximum overlapping between the Slepian window and the LSH, as proposed in this paper.

In this particular case, the optimal Slepian window has been achieved for $B_{W}/T_{W}=54.35$ Hz/s. The validity of this particular choice and the sensitivity of the method to variations of this parameter can be assessed measuring the entropy of the current spectrogram obtained with different Slepian windows, because small entropy values correspond to good energy concentrations [79,80]. The entropy of the current spectrogram has been computed with the method presented in [51,81]. Figure 9 shows the entropy of the LSH analyzed with the Slepian window for $B_{W}\cdot T_{W}=8$ (level of energy concentration) and for different values of $B_{W}/T_{W}$, from 0 to 2000 Hz/s. As can be seen in Figure 9, the criterion used to select the optimal value of $B_{W}/T_{W}$ of the Slepian window, ${\left(B_{W}/T_{W}\right)}_{opt}=54.35$ Hz/s, corresponds indeed to the choice of the minimum entropy (maximum energy concentration) of the LSH representation in the TF plane. Besides, the entropy around the optimal value is a smooth curve, as can be seen in Figure 9. This indicates that the computation process of $B_{W}/T_{W}$ in Equation (28) can tolerate small errors in determining the value of $\rho_{fault}$, which depends on the $t_{s=0.5}$ value in Equation (35). In this way, in the case of motors whose speed cannot be measured, it is still possible to use an estimated value of the time corresponding to a slip of 0.5 p.u. ($t_{s=0.5}$), equal to half of the total duration of the start-up transient (Figure 3), without any noticeable performance degradation of the diagnostic process.

## 4. Experimental Validation on a High-Power, High-Voltage IM

**Figure 10:**
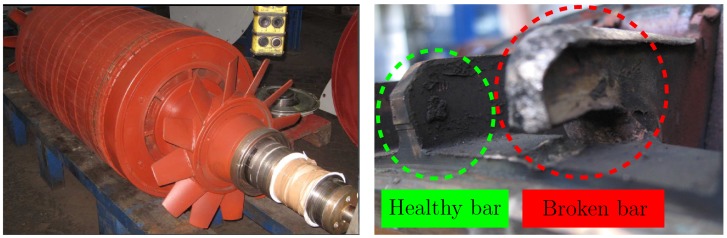
Rotor of the high-power, high-voltage IM given in Appendix B (**left**) and the detail of the broken rotor bar (**right**), used in the experimental validation of the proposed method.

**Figure 11:**
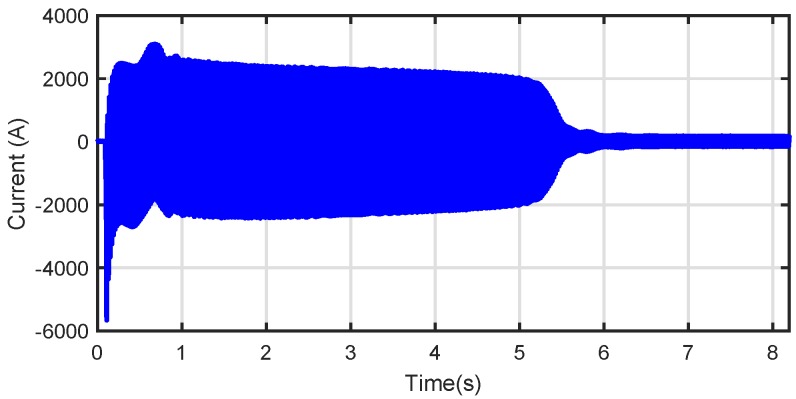
Stator current during the start-up transient of the high-power, high-voltage IM given in Appendix B with a broken bar fault.

The proposed method has been applied to the analysis of a high power (3.15 MW), high voltage (6 kV) IM working in an actual power plant, whose data are given in Appendix B. This IM has no sensor for speed measurement. The IM had a broken rotor bar, confirmed by visual inspection of the rotor (Figure 10). On the other hand, in the same factory, another IM of the same characteristics was installed. This second IM has not been reported regarding any anomaly and, thus, is meant to be in healthy condition. Nevertheless, it has never been subjected to a visual inspection of the rotor. The tests have been carried out during the start-up of the faulty and also of the healthy machine, powered directly from the mains ($f_{supply}\;=\;50\;\mathrm{Hz}$). The sampled current during the start-up of the faulty machine is shown in Figure 11. Both tests have been performed during 8.2 s using a sampling frequency of 6.4 kHz, with a total amount of 52,480 samples.

### 4.1. Choice of the Parameters of the Slepian Window for the Tested IM

The parameters of the Slepian window have been selected as proposed in Section 3. First, the value of the product $B_{W}\cdot T_{W}$ is selected to obtain a high energy concentration, so $B_{W}\cdot T_{W}=8$. Second, the ratio $B_{W}/T_{W}$ is set to be equal to the slope $\rho_{fault}$ of the LSH in the TF plane. For applying Equation (35), it is necessary to know the time when the slip reaches the value 0.5, $t_{s=0.5}$. In this case, as the speed is not measured, $t_{s=0.5}$ must be estimated. Nevertheless, as is shown in Figure 9, the entropy curve around the optimal value is smooth, so $t_{s=0.5}$ can be estimated as half of the total start-up transient duration Equation (34), without penalizing the proposed diagnostic procedure. Applying this criterion to Figure 11 gives $t_{s=0.5}≃3s$. Hence:

(36) $$\left.\begin{array}{ccc} B_{W}\cdot T_{W}= & 8\\ \frac{B_{W}}{T_{W}} & = & \frac{f_{supply}}{t_{s=0.5}}=\frac{50}{3} \end{array}\right\}\to \left.\begin{array}{ccc} B_{W} & = & 11.55\;\mathrm{Hz}\\ T_{W} & = & 692.8\;\mathrm{ms} \end{array}\right.$$

**Figure 12:**
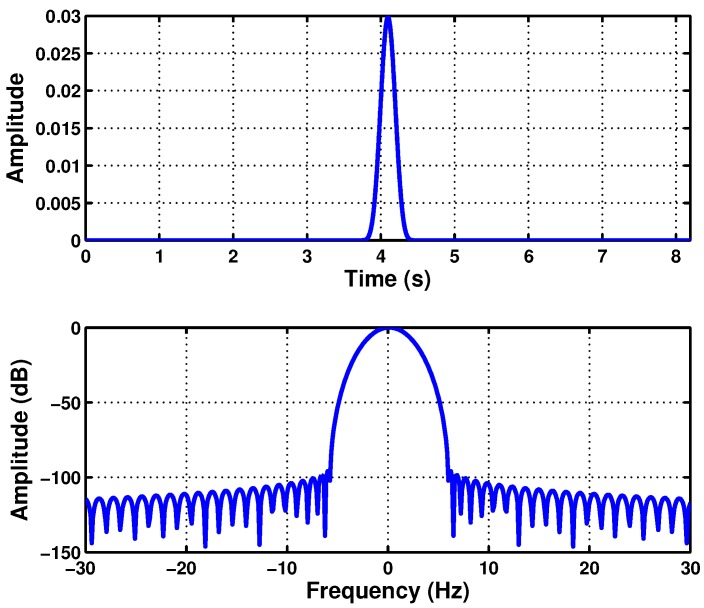
Slepian window ($B_{W}=11.55$ Hz, $T_{W}=692.8$ ms), optimized for detecting the LSH during the start-up of the high-power, high-voltage IM given in Appendix B, represented in the time (**top**) and in the frequency (**bottom**) domains.

**Figure 13:**
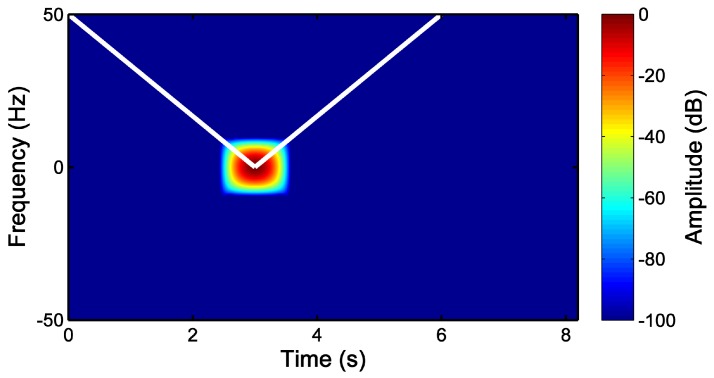
Heisenberg’s box of the atom of the Slepian window ($B_{W}=11.55$ Hz, $T_{W}=692.8$ ms), optimized for detecting the LSH during the start-up transient of the high-power, high-voltage IM given in Appendix B. The white line marks the estimated trajectory of the LSH in the time-frequency plane.

Figure 12 shows the Slepian window designed in separated time and frequency domains. In Figure 13, an atom of the Slepian window and the trajectory of the LSH are drawn in the TF plane. As can be seen, this window shape achieves the maximum overlap with the LSH trajectory, which coincides with the diagonal of the Slepian window.

### 4.2. Application of the Slepian Window to the Fault Diagnosis of the Tested IM

**Figure 14:**
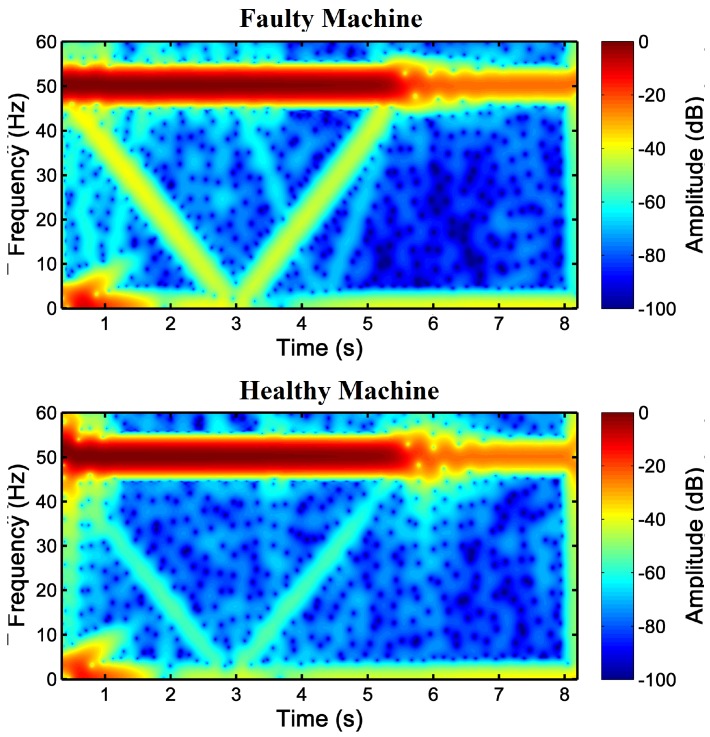
Spectrogram of the stator current computed with the proposed Slepian window, optimized for detecting the LSH during the start-up of the high-power, high-voltage IM given in Appendix B, with a broken bar (**top**) and in healthy conditions (**bottom**).

**Figure 15:**
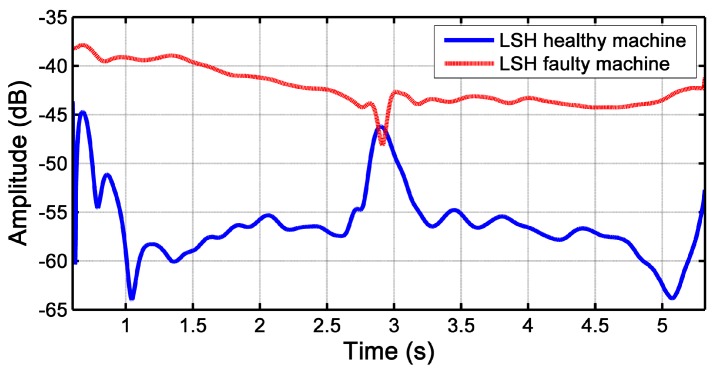
Amplitude of the LSH due to the broken rotor bar during the start-up of a healthy and faulty machine extracted from Figure 14. The average value of the LSH of the healthy machine (blue line) is −56.36 dB and of the faulty machine (red line) is −41.67 dB.

After the selection of the parameters of the Slepian window, it has been applied to the STFT of the motor stator current, to obtain the spectrograms shown in Figure 14 for both the faulty and the healthy IMs. In these cases, as the mains component has a much higher value than the amplitude of the LSH, a logarithmic scale (dB) has been applied to the spectrogram. In Figure 14, the characteristic V-shaped signature of the LSH in the TF plane appears clearly for both IMs. Nevertheless, as expected, the amplitude of the harmonic component corresponding to a broken rotor bar fault is much greater in the case of the faulty IM (Figure 14, top) than in the case of the healthy IM (Figure 14, bottom), whose V-shape corresponds to its inherent asymmetry. Figure 14 gives a visual representation, which enables a qualitative diagnosis. The amplitude of the ridges of the LSH during the start-up of both machines has been represented in Figure 15, to add a quantitative criterion and to improve the reliability of the diagnosis. In this figure, it can be seen that the LSH of the faulty machine has a greater amplitude (more than 10 dB) than the LSH of the healthy machine. Additionally, the average values of the LSH have been computed in healthy and faulty conditions. In the case of the healthy machine, the average amplitude of the LSH is −56.36 dB, whereas in the case of the faulty machine, it is −41.67 dB, which corresponds to a higher level of energy that confirms the presence of the fault.

It is worth mentioning that, as shown in Figure 15, for a healthy machine, the fault harmonics corresponding to a broken bar fault are present, due to constructive asymmetries, but they have a much lower value than for a faulty machine. In the case that no data are available about the machine in the healthy state, the MCSA method can be also applied, comparing the amplitude of the fault harmonics with a threshold value that indicates the presence of the fault. Threshold values have been obtained in the technical literature from the analytical analysis and experimental tests of the machine under faulty conditions [16], for harmonic components with frequencies given by Equations (1)–(3).

In the case of a broken bar fault, it is acknowledged that an amplitude greater than −45 dB of the main fault harmonic, k=±1 in Equation (1), is a clear indication of the presence of the fault. In the case of Figure 15, the value obtained is −41.67 dB, greater than the threshold value, which is a clear indicator of a broken bar fault, even in the case of not having data about the machine in healthy conditions.

## 5. Cost-Effective IM Fault Diagnosis Using the Truncated Slepian Window

In fault diagnostic systems, the spectrogram of the current is not computed on the continuous TF domain, as indicated in Equation (5), but on a discrete grid of points of the TF plane, as

(37) $$P_{SP}\left(m\cdot \Delta T,n\cdot \Delta F\right)={\left|S_{f}\left(m\cdot \Delta T,n\cdot \Delta F\right)\right|}^{2},\;n,m=0,1,2,3,\ldots .$$

In fact, the current signal is a discrete sequence, which is acquired sampling the stator current at a frequency $F_{sampling}$ during an acquisition time $T_{s}$. Therefore, the densest grid where the current spectrogram can be calculated using Equation (37) corresponds to a value of $\Delta T=1/F_{sampling}$, that is computing the FFT for every sample of the current, and to a value of $\Delta F=1/T_{s}$, that is using a window with the length of the current signal. This gives a total number of successive FFTs to be computed equal to $T_{s}\times F_{sampling}$, each one of length $T_{s}\times F_{sampling}$ samples. All the examples presented in the previous sections have been computed using this dense grid.

From a practical point of view, this election of $\Delta T=1/F_{sampling}$ and $\Delta F=1/T_{s}$ in Equation (37) is not the most adequate, because with these values, the computing time and memory resources needed to obtain the current spectrogram are very high. For example, it takes 154 s and 186 Mb to obtain each of the current spectrograms shown in Figure 14 on a personal computer (see Appendix C), which makes it difficult to implement this diagnostic technique in low power or embedded devices such as FPGAs or DSPs. To alleviate this problem, the spectrogram of the current signal can be obtained with a window shorter than the current signal, which reduces the length of the FFTs that must be performed at each time instant. Besides, since the local Fourier spectrum averages frequency variations taking place in the analysis window, it is not necessary to compute the successive FFTs for every sample of the discrete-time current signal, but they can be computed with some displacement [70]. Therefore, decimation in time and in frequency is almost always performed [70] when computing the current spectrogram. Therefore, a practical question is to find the minimum acceptable window length and the maximum acceptable shifting time that provide a high resolution diagnostic spectrogram of the stator current, keeping at a minimum the effort needed to obtain it.

This question does not have a simple answer in the case of a Gaussian window. The use of a window shorter than the current signal in the TF analysis has been seldom applied, due to the increase in bandwidth of the truncated window, which blurs the current spectrogram, rendering it useless. Some authors have proposed truncating the Gaussian window when its value falls below a given threshold, such as 0.01% of its maximum value, or using a truncated window with a length equal to six-times the standard deviation of the full-length window, $6\times \sigma_{t}$. Instead of truncating the Gaussian window, some authors propose using an efficient computation of the DGT with the full-length Gaussian window, based on a factorization algorithm [82,83,84], but this approach has a low penetration in the fault diagnosis field.

In this work, and thanks to the particular properties of the Slepian window (almost compact support both in the time and frequency of the discrete window), this problem is solved easily using an innovative and very cost-effective approach:

- Reducing the length of the FFT to the time duration $T_{W}$ of the Slepian window in Equation (35), much smaller than the length of the current signal $T_{s}$; that is, using a truncated Slepian window with a length equal to $T_{W}$, instead of the length of the current signal. This is equivalent to setting $\Delta F=1/T_{W}$ in Equation (37).
- Increasing the time shift of the window in successive FFTs to a value of $1/B_{W}$, where $B_{W}$ is the frequency bandwidth of the Slepian window in Equation (35), much longer than the time step between consecutive samples of the current, $1/F_{sampling}$. That is, setting $\Delta T=1/B_{W}$ in Equation (37).

The results obtained with the proposed approach are summarized in Table 1 and particularized in Table 2 for the example presented in Section 4. A huge reduction of the computational resources needed to obtain a diagnostic spectrogram when the proposed approach is used can be observed in this table. The time needed for computing the spectrogram has been reduced from 154.65 s to just 0.59 s (a 0.38% of the original time) and the amount of memory from 186,608 kB to just 59 kB (a 0.03% of the original memory usage).

**Figure 16:**
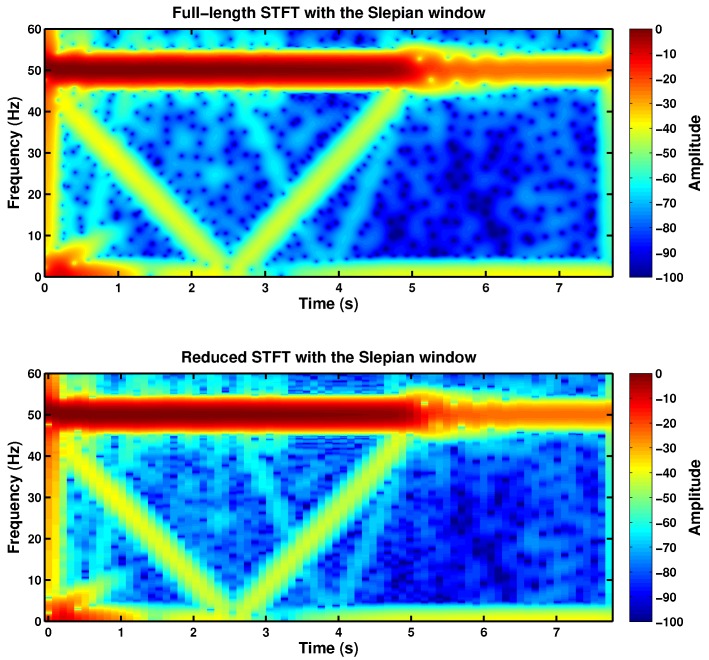
TF distribution of the stator current of the faulty machine presented in Section 4, using the full length TF analysis with a Slepian window (154.65 s, 186608 kB) (**top**) and using the proposed reduced length TF analysis with the truncated Slepian window (0.59 s, 59 kB) (**bottom**).

Figure 16 shows the spectrogram of the current of the faulty machine presented in Section 4, obtained using the traditional spectrogram (Figure 16, top), with the length of the Slepian window equal to the length of the current signal, and using the proposed decimated spectrogram (Figure 16, bottom), with a truncated Slepian window. Although the computing time has been greatly reduced to a 0.4% of the original time, the resultant spectrogram still shows clearly the LSH component generated by the fault.

### 5.1. Comparison between the Spectrograms Generated with the Truncated Gaussian Window and with the Truncated Slepian Window

**Figure 17:**
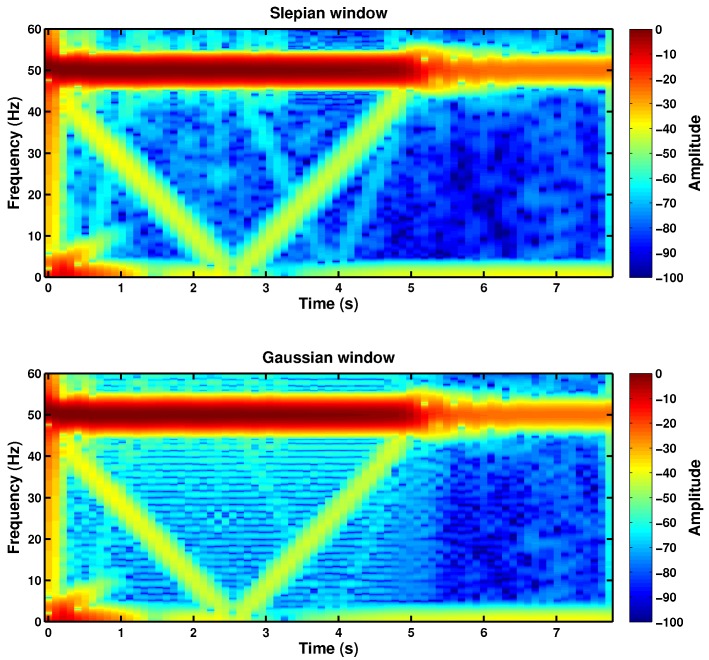
Reduced spectrogram of the high-power, high-voltage faulty machine given in Appendix B with a broken bar during the start-up transient using the truncated Slepian window (**top**) and using the truncated Gaussian window (**bottom**).

For comparison purposes, the spectrogram of the current of the faulty machine has been computed also with a truncated Gaussian window, using the values of window’s length and time shift obtained in the design of the truncated Slepian window presented in Table 2. Figure 17 shows that, for the same length, the truncated Slepian window (Figure 17, top) generates a current spectrogram much less blurred than the spectrogram generated with the truncated Gaussian window (Figure 17, bottom), thanks to its greater energy concentration. In fact, in the spectrogram generated with the truncated Slepian window, it is even possible to observe the signature of higher order fault harmonics (the V-shape with the vertex at *t* = 4 s), which are nearly indistinguishable in the spectrogram generated with the truncated Gaussian window. This increased resolution allows for a more accurate assessment of the motor’s condition.

## 6. Conclusions

TMCSA methods can extend the field of application of traditional MCSA methods to the fault diagnosis of electrical machines working in transient conditions, such as the start-up transient of an IM, by replacing the FFT with the STFT, which is able to display the signature of the fault components in the TF domain.

Traditionally, a gated Gaussian window has been used to perform the STFT, because an infinitely long Gaussian pulse achieves the minimum value of Heisenberg’s uncertainty principle. However, in this paper, it has been highlighted that there is a special function type, the Slepian function, which achieves the highest energy concentration for a finite duration and a finite bandwidth. Moreover, its atoms have a rectangular shape in the TF plane. Both features improve the resolution of the current spectrograms, highlighting the fault components and enabling more reliable diagnostic results. Besides, from a practical point of view, an important reduction in terms of computing time and memory resources can be achieved limiting the Fourier analysis to the length of the Slepian window and shifting the window in time steps equal to the inverse of the bandwidth of the Slepian window.

In this paper, the use of the Slepian window to perform the TMCSA of electrical machines in the transient regime has been proposed, for the first time to the best of the authors’ knowledge. The procedure for selecting the parameters of the Slepian window, depending on the type of the fault, has been also established and validated both with a synthetic fault component and with the tested current of a high-power, high-voltage IM with a broken bar. In future works, the proposed approach will be applied to the detection of other types of faults such as eccentricity or bearing faults.

## Figures and Tables

**Table 1:** Comparison of the parameters of the STFT of the current signal using the traditional full-length analysis and the proposed reduced length TF analysis, where $T_{s}$ is the length of the current signal, $F_{sampling}$ is the sampling frequency and $T_{W}$ and $B_{W}$ are the parameters of the Slepian window obtained from Equation (27).

	Full-Length TF Analysis	Reduced Length TF Analysis
Window duration (s)	$T_{s}$	$T_{W}=8/B_{W}$
Shift step (s)	$1/F_{sampling}$	$1/B_{W}$
FFT length (samples)	$T_{s}\cdot F_{sampling}$	$T_{W}\cdot F_{sampling}$
Number of FFTs	$T_{s}\cdot F_{sampling}$	$T_{s}\cdot B_{W}$

**Table 2:** Comparison of the parameters of the STFT of the current signal using the full-length and the proposed reduced length TF analysis, applied to the example presented in Section 4, where $T_{s}$ is the length of the current signal, $F_{sampling}$ is the sampling frequency and $T_{W}$ and $B_{W}$ are the parameters of the Slepian window obtained from Equation (27).

$T_{s}=8.2$ s , $F_{\mathrm{sampling}}=6.4\;\mathrm{kHz}$, T=0.6928s and B=11.55Hz		
	Full-Length TF Analysis	Reduced Length TF Analysis
Window’s length (s)	8.2	0.6928
Shift step (s)	$1.56\times 10^{-4}$	0.087
FFT length (samples)	52,480	4434
Number of FFTs	52,480	95
Time needed for computing the spectrogram (s)	154.65	0.59
Memory needed for computing the spectrogram (kB)	186,608	59

## Appendix A Simulated IM

Three-phase induction machine. Rated characteristics: P=1.1kW, f=50Hz, U=230/400V, I=2.7/4.6A, n=1410rpm, cosφ=0.8.

## Appendix B Industrial IM

Three-phase induction machine, star connection. Rated characteristics: P=3.15MW, f=50Hz, U=6kV, I=373A, n=2982rpm, cosφ=0.92.

## Appendix C Computer Features

CPU: Intel Core i7-2600K @ 3.40 GHz; RAM memory: 16 GB; MATLAB version: 9.0.0.341360 (R2016a).
